# Diphtheria

**Published:** 1859-10

**Authors:** W. Godfrey Dyas

**Affiliations:** Chicago


ARTICLE III.

### DIPHTHERIA.

BY W. GODFREY DYAS, M.D., CHICAGO.

In the year 1821, a memoir was read to the Royal Academy of Medicine, Paris, by M. Bretonneau, in reference to an epidemic disease which had made its appearance in the neighborhood of Tours. It was first observed in 1818, amongst some soldiers recently arrived from the Isle of Bourbon, but having been mistaken for scurvy, was unsuccessfully treated. From this date to the year 1826, other memoirs were presented to the Academy by M. Bretonneau, detailing the result of further investigation; and in the month of June of the same year, he published his celebrated work, entitled, "Des Inflammations Speciales du Tissu Muqueux, and en particulier de la Diphtherite ou Inflammation pelliculaire connue sous le nom de Croup, d' Angine Maligne, d' Angine Gangreneuse," etc.

In the meantime, observations embodying similar views were published in Great Britain, the enquiry having probably been suggested by Bretonneau's first memoir. But though among modern observers the claim to priority rests with him, yet we are not to infer the disease was unknown to the ancients. It would appear Hippocrates was not unacquainted with it, and one of the best descriptions of it has been given by Aretœus.

It has been termed diphtheria, or diphtherite, from the Greek *diphthera*, pellis, the formation of a pellicle or false membrane being invariably a symptom of the disease.

About one year before the publication of Bretonneau's work, Dr. Gregory, of London, made known the particulars of three cases of what he considered croup, wherein the disease having commenced in the fauces, passed downwards to the larynx and trachea, as far as the first division of the bronchial tube. The three cases were fatal. Two of them died from the blocking up of the larynx and trachea, with pseudo-membranous exudation, and one from exhaustion, as we are told, but the post-mortem examination seems not to have been a very searching one. At all events, the child was under the mercurial treatment, vigorously administered, and though no concretions were seen on opening the trachea, yet this was inflamed, and a purulent secretion covered its mucous membrane. These three cases occurred in the same family, and there was reason to suppose that the disease was communicated from one to another.

Thus, two important facts were brought to the notice of the profession,—one consisting in the contagious nature of the malady, the other in the progress of its local manifestation from above downwards.

The mere title of Bretonneau's work will at once show that he confounded the disease in question with croup; but he not only asserted the identity of these two, he also looked on angina maligna, not as similar, but even as identical with what was usually described under these several names.

Diphtheria is not the only disease whose early history tells of ill-defined terms—relations assumed from too limited a sphere of observation, and deductions in accordance—not true to all the cases they were meant to refer to. The same have been an episode in the history of every disease. The literature of our profession teaches how measles, scarlatina and small pox were confounded together; scarlatina described under the head of purpura; and roseola represented as a variety of rubeola.

We now proceed to give Bretonneau's description and treatment, and we will then enquire to what extent his views, under the aspects we have more recently seen the disease, correspond with the experience of later observers.

He describes it as usually commencing in one tonsil, seldom in both; fever slight; then white spots are seen on the surface of the affected tonsil; the cervical glands enlarge; deglutition not very difficult; redness surrounds the concretion, and it spreads rapidly to the velum palati, uvula, the other tonsil and the pharynx. The swelling of the lymphatics either subsides or remains stationary. After some hours or even days, a ringing cough, dry or accompanied by a frothy expectoration, announces the extension of disease to the respiratory organs. The following were the anatomical characters: Irregularly-shaped patches of redness, dotted and without swelling, coated with a concrete exudation; the most florid of these patches owing their color to a fine vascular injection. One or more long, narrow, red streaks were seen extending to the pharynx or descending to the trachea. A stripe of concrete matter on the centre of each of these streaks, and in the substance of these incipient concretions small semi-transparent bullæ are often seen. The edges of the pellicle were gradually confounded with the surrounding mucus, which, without being altered in its appearance, was changed in its quality, being no longer viscid, but coagulated near the concretion. These bands or stripes having extended, became more dense and homogeneous, and finally terminated by forming a complete tube, united to the subjacent mucous membrane by little prolongations that dipped into the mucous follicles. If the concretion were detached, the redness of the mucous membrane increased, a false membrane was reproduced, and in proportion as the super-imposed laminæ added to its thickness, it became more and more adherent to the surface beneath. Twenty-two dissections were made to ascertain if the concretion was the same as that of croup, and whether the mucous membrane beneath retained an unbroken surface. The result induced Bretonneau to infer that the exudation was identical with that of croup, and that, with some slight and occasional erosion excepted, the subjacent tissues preserved their integrity.

"In comparing," he writes, "the morbid appearances found on dissection in 55 bodies, of every age, who in the course of two years fell victims to epidemic angina, there appeared but one instance wherein the false membrane was confined to the trachea, without any similar exudation on the tonsils or other part of the pharynx. In no case, even of the most malignant nature, was there anything like gangrene of the parts. Ecchymoses of small extent, and an occasional slight erosion of those surfaces where the disease had longest continued, were the gravest alterations of structure which were ever seen. In about six or seven cases—that is, in one case in nine—the concretion extended to the ultimate ramifications of the bronchial tubes. In about a third of the whole number it reached the great divisions of the bronchia; in all the rest it terminated at various depths in the trachea, the mechanical obstruction of the respiration appearing constantly to be the immediate cause of death."

We are also told the concretions generally covered the pituitary membrane, but seldom extended to the external openings of the nostrils. In two cases the membrane lined the œsophagus, as far as the cardiac orifice of the stomach.

Bretonneau had no confidence in the curative powers of nature in this disease. He principally depended on the energetic application of concentrated muriatic acid, which he cautions us not to apply too frequently; and in case of the extension of the complaint to the larynx and trachea, his chief reliance then was on calomel and mercurial frictions,—the former administered in doses of three grains every hour, and the latter employed every third hour to the neck, arms and chest. As a last resource, he performed tracheotomy, by which, previously to the publication of his book, he had succeeded in saving one life out of three, the number operated on by him up to June, 1826.

During epidemics of diphtherite, Bretonneau and Trousseau saw the disease not confined to the fauces and respiratory passages, but extending to the skin, which was covered with false membranes. Trousseau allows that Bretonneau's observations, in reference to the pharyngeal symptoms being occasionally accompanied by a similar lesion of the skin, had been anticipated by others, but maintains that it was Bretonneau who first suitably arranged facts in connection with this disease, and completely described its symptoms.

We give Bretonneau's description and treatment of disease at some length, as we are aware that many do not suppose the diphtheria recently prevalent in England and some parts of this country to be the same as his diphtherite; and they rest their conjecture on the fact, that the course and termination of the disease as lately seen are not identical with those described by him.

We cannot deny that the progress and mode of termination of the several epidemics of diphtheria have varied more or less in every visitation of the calamity. But in this there is nothing unlike what we observe in the history of other epidemic diseases: Cholera, scarlatina, influenza and typhus can furnish evidence to show that their several epidemics vary not only in the intensity of development, but in the order of sequence of their symptoms, in the absence of some from the group, and in the appearance of others. The influenza of 1848, in Paris, was not the same as in 1831–37. The cholera of 1849, in England and Ireland, was unlike that of 1832. It had lost much of its severity—the form was not so spasmodic; it was rather the collapsed kind, and neither vomiting nor diarrhœa was so general. Nothing can be more variable than the progress and mode of termination of cases in the several epidemics of typhus: the morbid phenomena in one epidemic will be principally referrible to the brain and its membranes, in another to the respiratory organs, and in a third to those of digestion.

We will not now stop to discuss this question, but proceed to consider diphtheria both as a sporadic and epidemic disease, tracing it as observed at different periods, under different phases, and with varying severity, and show that though its essential characters were never wanting, yet incidental circumstances often associated themselves, with the effect of masking its main features. As seen in its sporadic form, diphtheria appeared much modified—this, from analogy, we should be led to expect. In such cases, it rarely extended to the air-passages, and generally terminated favorably; occasionally, however, it proved fatal by terminating in pneumonia, which, commencing insiduously, made progress without exhibiting any decided symptoms, and destroyed the patient when apparently reaching convalescence. A most interesting case illustrative of this has been related by Baudelocque, which we give at length:

"Victorine Soret, aged 13 years, strongly made, with a well-developed muscular system, working and sleeping for seven months in a damp work house, situated below the level of the street, in the quarter of La Greve, was seized early in Nov., 1834, with malaise, pain in the back and chilliness. These symptoms continued three days, when fever developed itself, accompanied with great difficulty of deglutition, swelling of the cervical glands, and nasal intonation of voice. A physician was called in, who applied thirty leeches to the angle of the jaws; at the same time, he prescribed emollient gargles, poultices and pediluvia. Two days afterwards he took blood from the arm. Under his treatment, there was no change for the better in the state of the throat; her strength was failing and her pulse becoming weak, and she fell into an adynamic state. It was now the fifteenth day of the disease, when she was taken to the Hopital des Enfans Malades. She presented the following symptoms: Nov. 26. Position, supine; face of a violet hue, with an expression of prostration and stupor; voice, nasal; tumefaction of the sides of the neck; occasionally, a return of fluids by the nares, which, at the same time, are the seat of a yellowish serous discharge of a sickening odour. On examining the throat, both tonsils, the pharynx and the uvula were seen covered with a greyish white false membrane, resembling gangrene. The breath was fetid; spitting abundant and sanious; tongue dry and like a piece of parchment; the abdomen free from pain; the skin rather cold than warm; pulse, from feebleness, cannot be reckoned; no pain of the chest; the patient, whose intellect is clear, assures us she has no local pain. (Cauterization of the pharnyx and tonsils with solution of nitrate of silver, four times; gargle of decoction of bark and chloride of soda; julep with 6 grs. of sulph. quinia; lemonade with wine). 27th. The same local and general symptoms persist; throat cauterized with a still more concentrated solution of nitrate of silver. The discharge from the nares indicate the extension of pellicular inflammation to the nasal fossæ, to these powdered alum was applied by means of a soufflоir. The rest of the treatment as on yesterday. 28th. Part of the exudation detached from the tonsils, but these remain swelled, presenting a brownish colour, and a rough, unequal surface; the difficulty of deglutition remains; no pain is complained of in the throat; the pulse can scarcely be felt; the same look of prostration and the same depression; two or three loose discharges from the bowels without pain. (Remedies as before, with the addition of blisters to the thighs). 29th. Some shreds of false membrane of a greenish yellow colour still adhere to the pharynx and left tonsil; the surrounding mucous membrane livid; vomiting took place during the visit; the vomited matter ejected partly by the mouth and partly by the nasal fossæ; a violent shivering in the afternoon of each of the last two days; to prevent a return, 12 grs. of sulph. quinia prescribed. 30th. The rigor returned in the evening; it has been of less continuance than on the preceding days; prostration increasing; the face is of a leaden hue; the tongue black, patient unable to sit erectly; skin cold; pulse thready, and but 64 pulsations in the minute; 20 inspirations, in the same space of time, can be counted. The blisters have scarcely reddened the skin; the examination of the fauces induces vomiting; the matter vomited contains the *debris* of false membranes; some greyish points exist on the pharynx and velum palati; the left tonsil covered over with a membraniform and very extensive concretion. Two liquid motions without pain; slight numbness of the limbs; intellect clear; pupils natural; no headache. From the 1st to the 5th Dec. Sinking more and more visible; pulse thready, and varying from 64 to 72 pulsations, and only on the 5th a notable acceleration; constant moaning; tongue pale and cold; more false membranes in the fauces, but the parts they cover are of a livid hue; diarrhœa; slight cough; no pain of the chest whatever. (Sinapisms to the inferior extremities. Lemonade, with wine, and preparations of bark internally). Death took place on the 5th.

"*Post-mortem examination nineteen hours after death.*—No cadaveric rigidity; no abnormal tumefaction of the velum palati, tonsils or pharynx; their surface of a deep red, and covered, in some points, with simple mucus, which the pulp of the finger can remove; mucous membrane not destroyed in any point; neither ulceration nor slough; no gangrenous odour. The epiglottis and the circumference of the glottis present the same redness as the pharynx; mucous membrane of the larynx, trachea and œsophagus pale; that of the bronchia red, the redness of which increases as the termination of the air tubes is approached; the cervical and bronchial glands hypertrophied; the pleuræ not the seat of any pseudo-membranous exudation; no fluid in their cavities; the superior and middle lobes of right lung crepitating and rose-colored externally; inferior lobe altogether impermeable to air, heavy and compact; when cut in slices, these fall to the bottom of a vessel with water. The inferior left lobe presents the same alteration posteriorly. Nothing remarkable about the heart and pericardium. *Abdomen.*—The gastric mucous membrane in contact with a brownish liquid; capillary system exhibiting some patches with an arborescent arrangement, and nipple-like eminences around the pylorus; its consistence has undergone no alteration; some redness from space to space along the intestinal canal, without any diminution or increase of the normal consistence of its mucous membrane; no alteration of Peyer's or Brunner's glands, which are scarcely visible; the other abdominal viscera gorged with blood, and less consistent than in the normal state. The head not examined."

We subjoin to this case some remarks by Baudelocque:

"This girl was admitted into hospital on the fifteenth day of the disease. An energetic antiphlogistic treatment had been administered, without improving the local affection. The exudation after having lined the velum, tonsils and pharynx, invaded the nasal fossæ, but did not extend to the respiratory passages. Whilst under the antiphlogistic treatment, symptoms of grave import supervened—the tongue became dry, the pulse was scarcely felt, and the skin was cold; patient fell into complete adynamia. Whilst in hospital, caustics were employed, the local symptoms seemed to give way, without any influence on the general system. In vain did they try to raise the strength by the aid of tonics, all were unavailing. Nothing during life revealed the pneumonia seen on the dead body. Cough was trifling, apparently caused by the detachment of the false membranes; respiration was not accelerated; no thoracic pain; no rust-colored expectoration. We do not hesitate to refer the lesion of lung to that hypostatic pneumonia which so frequently occurs in typhus fever, and which is but an element of a general morbid state. The general symptoms that preceded the invasion of diphtheria—those manifested during its course, and which persisted after its disappearance, leave no doubt that in this disease there is something besides the local lesion."

We cannot avoid thinking that many of the cases of diphtheria we have lately read, terminating in exhaustion, have proved fatal by latent pneumonia. The form of accompanying fever so resembles the typhus with which we find latent pneumonia frequently associated, as at once to suggest the probability of such occasional termination.

Though we detail this case with the view of not omitting anything that may tend to throw light on a subject that much needs elucidation, still it must be observed that in consequence of diphtheria, as a sporadic disease, not being very often met with, we must, in order to fully comprehend its nature, more fully study it in its epidemic form—that in which it presents itself most frequently, and affords the most favorable opportunities of our becoming acquainted with its characteristics.

During the years 1841, '42, '43 and '44, diphtheria prevailed as an epidemic in France, in the Department of the Saone and Loire, and La Nievere. The diphtheritic membrane was not confined to the mucous membrane, but extended to the cutaneous surface, yet in varying proportions. The pharyngeal form was the most frequent; next in order of frequency, the skin, the laryngo-tracheal and the buccal mucous membranes. These were instances of simple diphtheria confined to one situation. But in other cases, the pseudo-membranes formed simultaneously on many and distant points of the system; thus there were the pharyngo-cutaneous, the laryngo-cutaneous and the pharyngo-laryngeal diphtheria. In this epidemic the local seemed to precede the general symptoms: the face was much swollen; febrile reaction more or less intense; pulse in general very frequent, and almost always very hard and contracted; there was headache; the tongue was swollen and covered with a thick and yellowish coat; nausea and frequent vomiting; sometimes phlegmonous abscesses either in or around the conglobate glands. The disease reached its maximum of intensity in 36 to 48 hours. The tonsils became enormously enlarged, even becoming a mechanical obstacle to the passage of air or liquids. When the patients had been previously cachectic, the disease rapidly proceeded to a fatal termination, the pseudo-membranes having been brownish even from the commencement.

Most commonly, death from diphtheria took place from the seventh to the tenth day. When the disease was about to terminate favorably, the false membranes ceased to extend, became circumscribed by a red circle, assumed a swollen appearance, detached themselves in shreds accompanied by an oozing of blood, and were thrown off with a frothy fetid saliva. During this epidemic, the larynx and trachea were rarely affected by diphtheric inflammation. There were not many, therefore, affected with symptoms like croup; but as we have seen in some sporadic cases, so in the epidemic form, both Daviot and Guersent tell of unfavorable terminations by broncho-pneumonia, generally double, stealing imperceptibly on when the patient was either convalescing or health nearly re-established. Daviot does not think pharyngeal diphtheria contagious—an opinion in which few will concur. He thought the objects which the physician in his treatment should keep in view, were the subduing of inflammation, the destruction of false membranes and the modifying the special morbid state of the mucous membrane. For the first, he recommends general bleeding, which he supposed particularly suitable for all beyond 10 years of age, where the disease is in its early stage and the general reaction violent. With this he advised local bleedings to be conjoined, to be repeated at short intervals. He restricts the use of vomits to very young subjects. He objects to purgatives, as augmenting the predisposition to intestinal irritation, and as being without any advantage. He also objects to blisters, as having the serious inconvenience of adding cutaneous to pharyngeal diphtherite; he however approves of rubefacients. Alum he thought useful in the early stage, but useless afterwards, when he preferred nitrate of silver to any other local application, even to muriatic acid. Calomel he looked on as useless in pharyngeal, but particularly serviceable in cutaneous diphtherite. He mentions the following important fact to show identity of nature in pellicular inflammation of mucous membrane and of the skin. During this epidemic he saw, under the influence of similar causes, diphtheria rage in several members of the same family, attacking in the one the pharynx, in another the skin, in another the respiratory passages, and in another all these at once or successively. How will those who maintain that croup is essentially a distinct disease meet this fact? Daviot's monograph is considered as one of the best on this subject. He lost two children by the epidemic. This circumstance is supposed to have ensured unusual care and exactness in his observations and statements, as his essay was written, most probably, from impressions, unfading as they were sad, which memory must have vividly recalled whilst recording symptoms so painfully associated with domestic affliction.

(TO BE CONTINUED).

